# Letter to Editor regarding: “Atypical intradural extramedullary spinal schwannoma causing cauda equina syndrome: A case report and literature review”

**DOI:** 10.1016/j.ijscr.2023.108707

**Published:** 2023-08-22

**Authors:** Borislav Kitov, Atanas N. Davarski, Denis Milkov

**Affiliations:** a Clinic of Neurosurgery, University Hospital St. George, Plovdiv, Bulgaria; b Department of Neurosurgery, Faculty of Medicine, Medical University of Plovdiv, Plovdiv, Bulgaria; c Clinic of Otolaryngology, University Hospital Kaspela, Plovdiv, Bulgaria

Dear Editor,

We read with interest the article by Lim D.-J., published in your esteemed journal, reporting the case of acute development of cauda equina syndrome due to a schwannoma located on the level of L3 vertebra [1]. The article provoked some questions for us, to which we did not find answers in the publication. Based on our own experience and the literature data, we would like to share our contemplations with the audience of your journal.

Firstly, it is not clear what meaning the author places in his use of the term “atypical schwannoma”. The atypicality of a tumor concerns its histological structure or its location. Schwannomas originate from the Schwann sheaths of the spinal roots, which are outside the spinal cord, and several cellular types have been described: cellular schwannoma, plexiform schwannoma, and ancient schwannoma [1,2]. Schwannomas are tumors with a benign biological behavior. They are histologically classified as grade I according to the World Health Organization, and histological atypicality is very rarely observed [3]. Regarding the tumor location, it would be considered atypical if it were intramedullary, as such a location according to Koeller and Shih is observed in only 1.1 % of cases of spinal schwannomas [2]. The lumbar location of spinal schwannomas in their study was observed from 25.6 % to 60 % of cases, which according to Jinnai et al., is due to the anatomical characteristics of the spinal nerves, which travel a greater distance in the region of the cauda equina [4,5]. In this regard, the localization of the schwannoma described by Lim D.-J. can be considered typical. According to Fountain et al., the ancient schwannoma variant is extremely rare in the cauda equina region, but Lim D.-J. did not specify the histological variant of the schwannoma he excised in this region [6].

A significant part of the article by Lim D.-J. concerns the acute development of cauda equina syndrome, and it is important to consider possible causes for its occurrence. In our opinion, several aspects should be considered. The lumbar canal at the level of L3 is narrowest, with a sagittal diameter of 14.18 mm and width of about 21 mm in women [7]. According to Fujii et al., if the size of the tumor is >20 % of the axial size of the spinal canal and >40 % of the sagittal size, it causes clinical symptoms [8]. The size of the shwanomma removed by Lim D.-J. was 1.2/1.2 cm, which represents filling of 85 % of the sagittal dimension and 57 % of the axial dimension of the canal on the level of the L3 vertebra, suggesting the development of significant stenosis and compression of the cauda equina roots. On the other hand, it is known that schwannomas located in the region of cauda equina are relatively mobile, which allows for some rotation, as a result of which infarction and/or hemorrhage can occur, causing an acute onset of a neurological deficit [9]. The finding by Lim D.-J. of a clot passing through the affected nerve root supports this thesis.

Despite some terminological differences with the authors, we must admit that the article is valuable because it shows that a slow-growing, benign tumor such as a schwannoma can, under certain conditions, cause a severe neurological deficit within a few hours.

## Consent

Consent is not applicable because this manuscript is a Letter to Editor.

## Ethical approval

Ethics approval is not applicable because this manuscript is a Letter to Editor.

## Funding

No sources of funding to declare.

## Author contribution

Author contribution is not applicable because this manucript is a Letter to Editor.

## Guarantor

Guarantor is not applicable because this manuscript is a Letter to Editor.

## Research registration number

Registration of Research Studies is not applicable because this manuscript is a Letter to Editor.

## Conflict of interest statement

No conflict of interest to declare.
